# Diagnosis of cardiac amyloidosis: a systematic review on the role of imaging and biomarkers

**DOI:** 10.1186/s12872-018-0952-8

**Published:** 2018-12-04

**Authors:** Panagiota Kyriakou, Dimitrios Mouselimis, Anastasios Tsarouchas, Angelos Rigopoulos, Constantinos Bakogiannis, Michel Noutsias, Vasileios Vassilikos

**Affiliations:** 1 3rd Cardiology Department, Ippokrateion General Hospital of Thessaloniki, Konstantinoupoleos 49, 55 642 Thessaloniki, GR Greece; 2 Mid-German Heart Center, Department of Internal Medicine III (KIM-III), Division of Cardiology, Angiology and Intensive Medical Care, University Hospital Halle, Martin-Luther-University Halle-Wittenberg, Ernst-Grube-Strasse 40, Halle (Saale), D-06120 Germany

**Keywords:** Amyloidosis, Cardiac amyloidosis, Heart failure, Echocardiography, Strain imaging, Biomarkers

## Abstract

**Background:**

Cardiac Amyloidosis (CA) pertains to the cardiac involvement of a group of diseases, in which misfolded proteins deposit in tissues and cause progressive organ damage. The vast majority of CA cases are caused by light chain amyloidosis (AL) and transthyretin amyloidosis (ATTR). The increased awareness of these diseases has led to an increment of newly diagnosed cases each year.

**Methods:**

We performed multiple searches on MEDLINE, EMBASE and the Cochrane Database of Systematic Reviews. Several search terms were used, such as “cardiac amyloidosis”, “diagnostic modalities cardiac amyloidosis” and “staging cardiac amyloidosis”. Emphasis was given on original articles describing novel diagnostic and staging approaches to the disease.

**Results:**

Imaging techniques are indispensable to diagnosing CA. Novel ultrasonographic techniques boast high sensitivity and specificity for the disease. Nuclear imaging has repeatedly proved its worth in the diagnostic procedure, with efforts now focusing on standardization and quantification of amyloid load. Because the latter would be invaluable for any staging system, those spearheading research in magnetic resonance imaging of the disease are also trying to come up with accurate tools to quantify amyloid burden. Staging tools are currently being developed and validated for ATTR CA, in the spirit of the acclaimed Mayo Staging System for AL.

**Conclusion:**

Cardiac involvement confers significant morbidity and mortality in all types of amyloidosis. Great effort is made to reduce the time to diagnosis, as treatment in the initial stages of the disease is tied to better prognosis. The results of these efforts are highly sensitive and specific diagnostic modalities that are also reasonably cost effective.

## Background

Amyloidosis refers to a group of diseases characterized by the deposition of amyloid fibrils in multiple tissues throughout the body, such as in the liver, kidney, eyes, heart and others. Amyloid fibrils result from the uncontrolled deposition of structurally abnormal proteins. Cardiac amyloidosis (CA) refers to the infiltration of the myocardium by amyloid fibrils, which cause cardiac dysfunction, eventually leading to heart failure [1]. The effect of amyloidosis on the quality of life and mortality rate of patients is substantial. A recent study enrolling patients with light chain amyloidosis (AL) found significant mental and physical impairment directly attributable to the disease [2].

Each disease that belongs to the umbrella term of amyloidosis is caused by copies of a specific protein that are folded in a fibrillogenic conformation. Not all types of amyloidosis affect the heart in the same frequency. Cardiac amyloidosis encountered in clinical practice is in the vast majority of cases caused by light chain (AL) or transthyretin amyloidosis (ATTR), with the latter consisting of two subtypes: Senile ATTR amyloidosis, which is caused by wild-type transthyretin deposition (ATTRwt), and familial ATTR amyloidosis, which is caused by mutant proteins that exhibit increased fibrillogenicity (ATTRm or ATTRv, where ‘v’ stems from ‘variant’) [1, 3, 4]. Secondary amyloidosis is the result of the overproduction of the acute-phase protein serum amyloid A (SAA) in chronic inflammatory conditions. SAA has been shown to deposit in cardiac tissue, but clinically significant cardiac involvement appears to be rare [5]. Isolated atrial amyloidosis (IAA) is a condition of great importance to the pathophysiology of arrhythmias that originate in the atria, such as atrial fibrillation [6]. IAA is caused by the deposition of Atrial Natriuretic Peptide (ANP) fibrils in the atria. It appears to be the most common type of amyloidosis to affect the heart, as more than 90% of people over 90 years old appear to have measurable ANP deposition in their hearts [7]. The disease’s predilection for older women appears to stem from the fact that estradiol upregulates ANP expression in atrial cardiomyocytes [8].

Systemic amyloidosis is a quite rare disease, with its incidence in the English population in the year 2008 estimated to be at least 4/1.000.000, as extrapolated from epidemiological data. Most of the patients affected were aged between 60 and 79 years [9]. The minimum incidence of AL is similarly estimated at 3/1.000.000, and the prevalence of AL CA, i.e. the cardiac involvement in AL, is estimated at 8–12/1.000.000 [10]. AL CA affects patients aged between 55 and 60 years old, with men appearing to be slightly more vulnerable to the disease [11]. On the other hand, ATTRwt CA usually affects older patients, with the results of autopsies showing that 25% of people older than 80 years have their myocardium infiltrated by TTR amyloid depositions [10, 12]. Interestingly, ATTRwt CA has been proved via 99mTc-DPD scintigraphy to represent 13% of patients with Heart Failure with a Preserved Ejection Fraction (HFpEF), in a sample of 120 patients over 60 years old [13]. CA is the primary cause of restrictive cardiomyopathy (RCM) [14, 15]. In summary, cardiac amyloidosis’ frequency among HF patients is increasingly being acknowledged by clinicians and researchers alike, as can be observed from its inclusion in the novel MOGES classification of cardiomyopathies [16].

In the latter stages, CA, as a typical example of restrictive cardiomyopathy, manifests with the classical triad of congestive HF symptoms, i.e. shortness of breath, fatigue and edema [1]. Cardiac pump dysfunction progressively emerges as amyloid aggregates in the heart tissue [17]. ECGs of patients already diagnosed with the disease are seldom normal, the most frequent abnormalities for both AL and ATTRwt CA being low voltage QRS and a pseudoinfarction pattern. Conduction disorders and arrhythmias are also common, especially atrial fibrillation and atrioventricular disorders [18, 19]. Nonetheless, amyloidosis is probably still under-diagnosed, especially in subsets of the population like elderly patients with HF [20]. Endomyocardial biopsy remains the gold standard of CA amyloidosis’ diagnosis [21]. Regarding AL, fat pad biopsy has been proven to have great sensitivity to confirm the diagnosis [22]. A quick and efficient diagnostic approach in CA is of great significance given the accelerated deterioration observed in advanced stages of the disease. It is known that the onset of a restrictive pathophysiology is independently linked to a significantly poor prognosis in CA [23]. Additionally, early diagnosis offers more therapeutic options, as advanced cardiac failure is a contraindication for other therapies such as orthotopic liver transplantation [24]. The diagnostic modalities that will be discussed below represent efforts to offer the clinicians with tools in order to minimize lost cases, significantly reduce the time needed for making the diagnosis, whilst simultaneously maintaining reasonable cost-effectiveness.

## Methods

Adhering to PRISMA guidelines and aiming to procure the latest literature on the subject of the methods used to diagnose cardiac amyloidosis, all studies would be evaluated for eligibility based on the following criteria: Only (1) original studies (2) published in peer-reviewed journals (3) within the past 5 years were considered eligible for inclusion in this systematic review. (4) Only studies written in English were evaluated for inclusion.

**Fig. 1:**
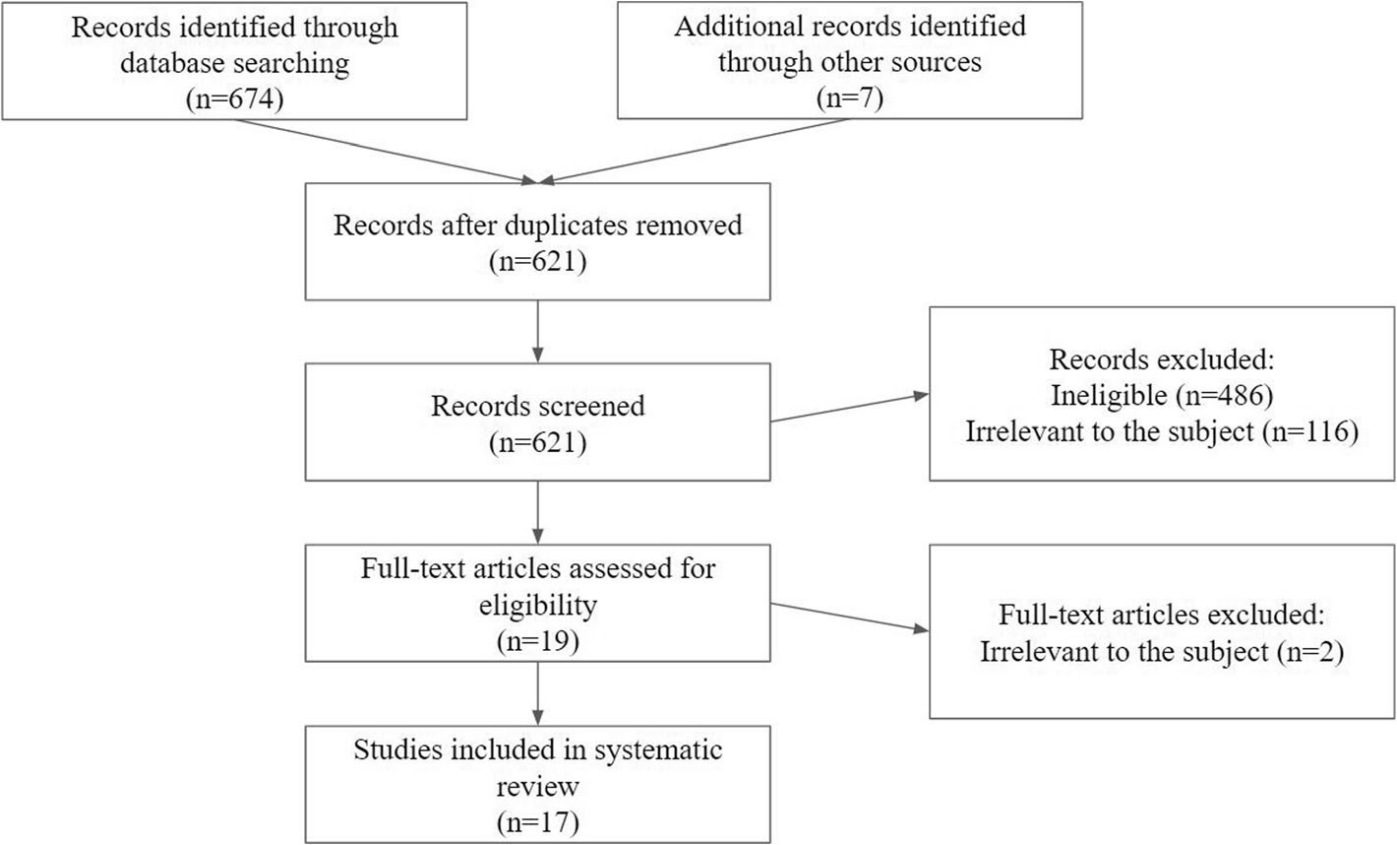
Flowchart of literature review process

Studies were looked up on MEDLINE, EMBASE, PMC and the Cochrane Database of Systemic Reviews through PubMed and Google Scholar. Several search terms were used, such as “cardiac amyloidosis diagnosis”, “staging cardiac amyloidosis”, “TTR amyloidosis imaging” and “cardiac amyloidosis ultrasound”, “cardiac amyloidosis GLS”, “cardiac amyloidosis EFSR”, “cardiac amyloidosis MRI”, “cardiac amyloidosis LGE”, “cardiac amyloidosis CMR”, “cardiac amyloidosis nuclear imaging”, “cardiac amyloidosis FDG-PET”, “cardiac amyloidosis PET”. All studies in the first three pages of results for each search query were evaluated for inclusion in this systematic review based on the aforementioned eligibility criteria. The search results that appeared eligible on the basis of their title, publication date and abstract were given full consideration and were included in the systematic review, provided a comprehensive examination of their full text confirmed their eligibility (Fig. 1).

## Results

The search that was conducted yielded 674 results, of which 60 were confirmed to be duplicates. Seven studies included were recommended by experts on the field. 621 records were screened on the basis of title and abstract, of which 602 were excluded from the review. Of the remaining 19 studies, 2 were not suitable for our review. The remaining 17 articles were finally included in the systematic review.

### Imaging methods

#### Cardiac ultrasound

Cardiac ultrasound is a widely available, easy to use, radiation-free and relatively inexpensive bedside tool. Cardiac ultrasound has been established as the first step in the amyloidosis diagnostic workup, as a tool of “ruling in” the diagnosis, identifying patients likely to have the disease and prompting further workup. Nowadays, advanced ultrasonographic protocols, utilizing state of the art ultrasound technology are hugely improving the method’s sensitivity and specificity.

The most common two-dimensional (2D) echocardiographic findings observed in CA are biatrial dilatation and increased left and right ventricular wall thickening [25, 26]. It should be noted that there is a mismatch between ECG and echocardiographic findings, as low QRS voltage is not consistent with ventricular hypertrophy. Granular sparkling appearance of myocardium is a common echocardiographic finding in CA, which is attributed to the increased echogenicity of the amyloid protein [25, 26]. Furthermore, valvular thickening could be also found in specific types of CA, such as TTR amyloidosis. However, it should be noted that the frequency of these findings increases at the later stages of the disease. Clinical data demonstrated the importance of a more multiparametric approach, including the evaluation of more advanced techniques such as Tissue Doppler Imaging (TDI) and 2D strain imaging. Findings of RCM are typical in CA. The severity of diastolic function is related to the degree of amyloid infiltration, while high filling pressures and restrictive mitral inflow pattern are also observed. At the initial stages, an abnormal relaxation pattern is observed. However, the increase of wall thickness with the progression of the disease leads to shortened deceleration time, high early velocity (E-wave) and low atrial velocity (A-wave) leading to E/A ratio > 2 and deceleration time < 150 ms, compared to E/A ratio < 1 at the early stages [23, 27, 28]. Peak early diastolic velocity (E’) assessed by TDI is decreased in the earliest stages of the disease, and further decreases with the disease progression, a fact that also helps in the differential diagnosis with other diseases, such as constrictive pericarditis or hypertrophic cardiomyopathy (HCM), in which E’ is normal or mildly reduced [29]. Furthermore, numerous studies confirmed that impaired longitudinal function assessed by TDI plays key role in the early diagnosis of CA. It has been demonstrated that basal and mid LV longitudinal myocardial deformation were significantly decreased in asymptomatic CA patients [27]. This abnormal finding was observed before wall thickening. Interestingly, it was shown that impaired longitudinal function assessed by TDI could discriminate between patients with CA and patients with amyloidosis without cardiac involvement [27, 28]. However, it should be noted that the TDI technique has significant limitations related to the Doppler effect as well the influence of noise and angle dependence on measurements. Beyond TDI, myocardial performance index (MPI also called Tei index), calculated by combining systolic and diastolic time intervals, could provide significant information on myocardial function of CA patients, while it could also serve as an outcome indicator [30, 31].

**Fig. 2:**
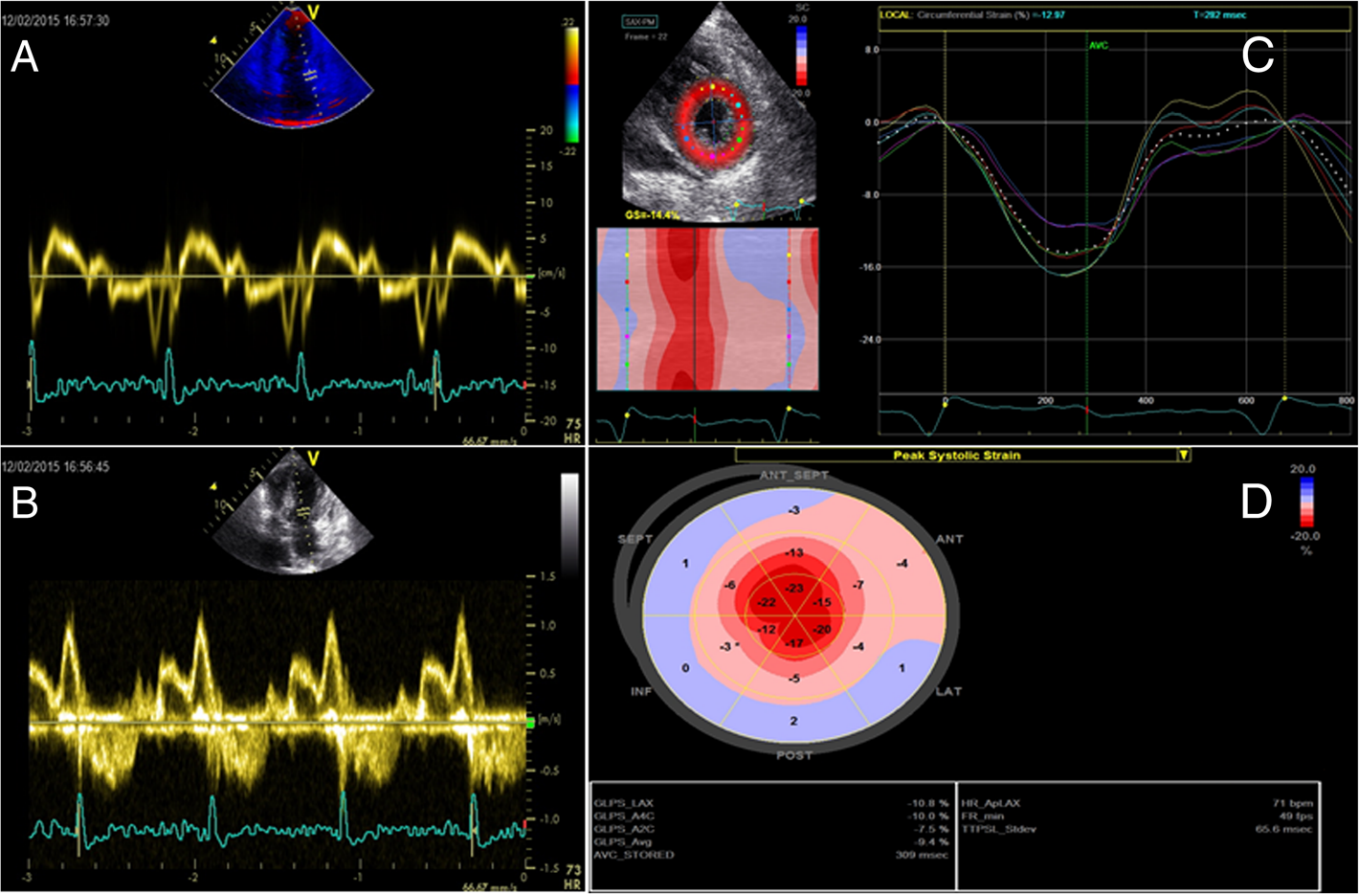
Mitral valve inflow pulsed wave Doppler (**a**) and Tissue Doppler Imaging of the mitral valve annulus (**b**) of a patient with CA demonstrating an early diastolic dysfunction pattern. Circumferential (**c**) and longitudinal deformation by 2D strain imaging (**c** and **d**) mainly shows an impairment of global longitudinal deformation. In patients with CA, an impaired deformation in basal segments compared to apical segments could be found

Speckle tracking technique for the evaluation of myocardial deformation helped us to overcome these limitations. Based on the interaction between ultrasounds and tissues and the use of specific software, two-dimensional speckle tracking (2DST) is able to evaluate longitudinal, radial and circumferential deformations. From the very first studies, it has been demonstrated that global longitudinal strain (GLS), circumferential and radial deformations were significantly decreased in CA patients, compared to patients with hypertrophic cardiomyopathy or hypertensive heart disease [32]. Notably, it has been reported that patients with AL and TTR cardiac amyloidosis and preserved ejection fraction (EF) had impaired basal and mid LV longitudinal strain (LS), also visible via TDI, while apical LS was preserved [33]. Further, Phelan et al. compared the global and regional strain parameters of 55 patients diagnosed with AL CA to that of 30 patients with HCM of different etiology. They used the parameter of relative apical longitudinal strain (RALS), average apical LS/(average basal LS + average mid-LS). An abnormally high RALS displayed a sensitivity of 93% and a specificity of 82% at detecting CA. Relative apical sparing is characteristic of both AL and TTR CA [34]. Pagourelias et al. used the ratio of GLS to the EF, which is characteristically disrupted by amyloid deposits. They introduced the ejection fraction strain ratio (EFSR) as a reliable tool to diagnose CA [35] and confirmed that it is currently the most specific (91.7%) and sensitive (89.7%) echocardiographic parameter in diagnosing CA. The apical sparing parameter proposed by Phelan et al. was also investigated in the study, but yielded a markedly low sensitivity of 37.5% in that sample population, utilizing the proposed cut-off values [36]. The study also confirmed that EFSR displays low inter-observer variability, being a standardized parameter, and demonstrably retains its high diagnostic value in populations with either increased wall thickness or preserved EF. Figure 2 is representative of echocardiographic findings in CA.

One of the recent developments is the clinical implementation of three-dimensional (3D) echocardiography and 3D speckle tracking (3DST) technique. In one of the first studies, LV regional dyssynchrony was measured via a 16-segment dyssynchrony index in AL patients and was found significantly impaired compared to control subjects [37]. In addition, very recent studies used deformation and rotational 3DST parameter in order to differentiate CA patients from patients with other forms of myocardial hypertrophy [37]. One of the studies has further confirmed with the use of 3DST that the basal rotational strain was significantly reduced compared to apical rotational strain, a finding that could efficiently differentiate CA patients from patients suffering from HCM [38]. However, it is important to mention that 3D echocardiography is still not widely used compared to 2D echocardiography in clinical routine. Further developments and clinical studies are needed in order to develop measures and techniques that could offer a significant additive value compared to 2D echocardiography.

#### Cardiac MRI

Cardiac Magnetic Resonance (CMR) is considered a very sensitive and specific diagnostic modality for both ATTR and AL, although it is more time-consuming and expensive when compared to cardiac ultrasound [39, 40]. CMR demonstrated high sensitivity in diagnosing further diseases, such as in myocardial iron overload [41], although cardiac ultrasound remains the first choice as it is easy to use and widely available, as demonstrated in studies which compared the sensitivity of normal echocardiography compared to CMR [42, 43]. The deposition of amyloid in the heart leads to an increase in myocardial extracellular volume (ECV). This increase is readily detected by CMR through the Late Gadolinium Enhancement (LGE) test. Gadolinium-based contrasts rapidly extravasate are not absorbed by healthy cardiomyocytes and is rapidly removed from the circulation, while any in the elimination of contrast indicates an increase in ECV, and it is this tissue that shows up positive in LGE tests CA characteristically leads to diffuse subendothelial LGE in the left ventricle as well as the atria [44].During the disease progression, LGE expands transmurally and gradually to all chambers of the heart tissue [45].

CMR with LGE demonstrated 80% sensitivity and 90% specificity in detecting cardiac involvement in amyloidosis [46]. Since then its predictive value has improved even further [47], in great part thanks to the development of phase-sensitive inversion recovery (PSIR). This technique, which allows the use of different inversion times, alleviates some previous technical problems of LGE CMR, which would cause discrepancies in LGE measurement, leading to an underestimation of LGE extent and severity in cases where the majority of cardiac tissue would be affected [48]. Based on the increasing accuracy CMR findings, many clinicians actually forgo the step of confirming the diagnosis through endomyocardial biopsy (EMB), when a typical LGE pattern is detected and no other causes of focal increase in cardiac ECV, such as myocarditis, are likely. Although it is difficult to assess the implications of this approach, the inclusion of CA patients in studies whose diagnosis has not been confirmed through EMB has been contested [39]. Furthermore, it has been claimed that CMR can also differentiate between ATTR and AL, due to differences in the pattern of LGE [49], although further investigation is necessary.

**Fig. 3:**
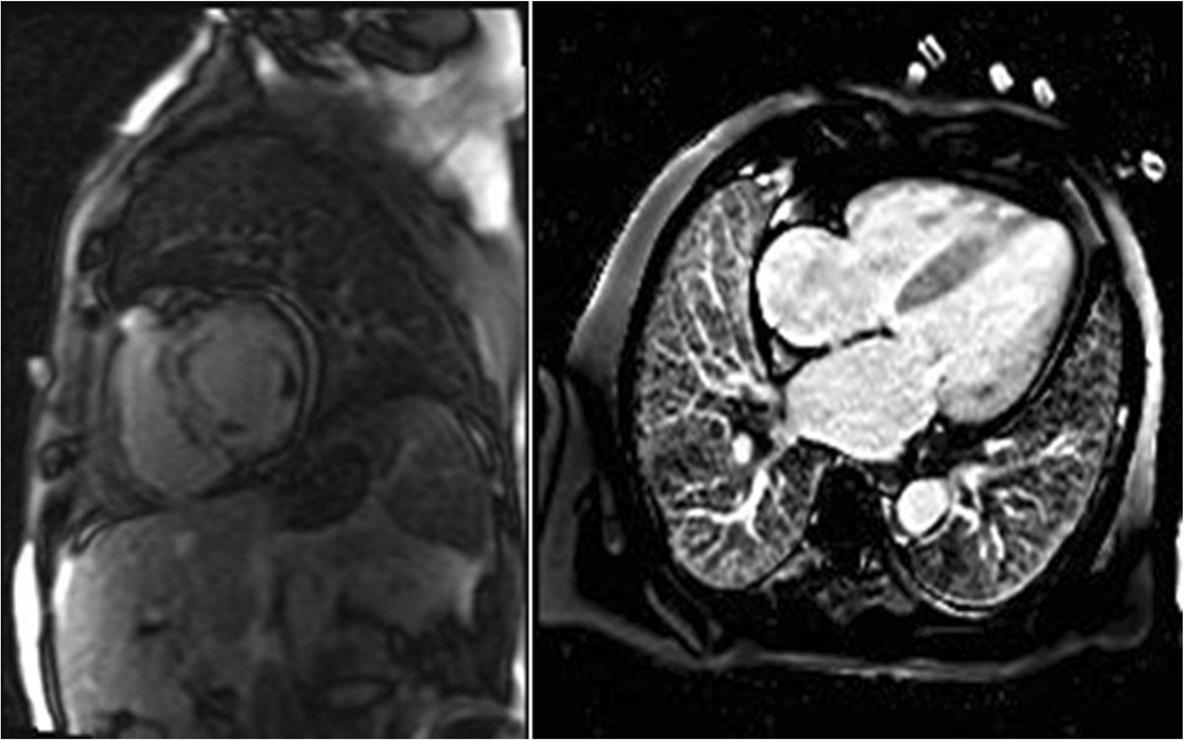
Cardiac magnetic resonance (CMR) images of patients with cardiac amyloidosis. Amyloid fibril deposition pattern mainly affects subendocardial CMR imaging, leading to a shortened T1 relaxation time and a diffuse LGE of the left ventricular endocardium

CMR’s role in the differential diagnosis between amyloidosis and other diseases such as Fabry disease, and HCM has also been examined. Specifically, while HCM shows a patchy LGE pattern mainly located in the middle of the hypertrophic wall and Fabry disease shows a more located LGE pattern in the basal segment of the LV, CA shows atypical signal intensity related to the amyloid deposition pattern and faster clearance of gadolinium [50]. Due to this faster clearance of gadolinium, CA patients also present a diminished T1 difference between myocardium and blood pool. However, because of the reduced specificity of CMR in distinguishing between the aforementioned diseases in some cases, the simultaneous examination of LGE and the relative apical strain sparing was necessary to yield the best results, being capable of very reliably detecting CA patients (Fig. 3) [51]. A technique that attempts to quantify the LGE findings, the Look-Locker magnetic resonance sequence, was tested in real-life patient series and yielded mixed results [52]. Higher T1 inversion times (roughly the time it takes for T1 signals to drop to pre-contrast levels) correlated with amyloid load but not patient prognosis.

Non-contrast T1 CMR imaging constitutes a very promising technique with a reported 92% accuracy in detecting cardiac involvement in amyloidosis. Notably, it is safe for patients with renal failure, which is a common problem among patients with amyloidosis [53, 54]. Efforts to quantify amyloid load using T1 images have yielded several techniques that boast reliable detection of increased ECV, which is tied to higher amyloid load in EMB specimens and worse patient outcome in small studies [55–57].

#### Nuclear imaging

Nuclear imaging of the heart pertains to the intravenous administration of radioactive substances and the interpretation its absorption throughout cardiac tissues by measuring the emitted radiation. Radio-labeled phosphonates, generally referred to as bone tracers, such as [99mTc]-DPD show strong affinity for TTR amyloid fibrils in cardiac tissue. This has been extensively utilized in the CA diagnostic process [58, 59]. The exact mechanism of [99mTc]-DPD binding to the amyloid fibrils is yet to be elucidated, although the higher calcium content of the TTR fibrils may play a decisive role [60]. [99mTc]-DPD scans showed a surprisingly high sensitivity and specificity approaching 100% for ATTR CA whilst being capable of very reliably excluding AL CA [61]. This latter finding has been somewhat contested, as AL amyloid deposits exhibiting uptake have been reported [62]. Its sensitivity in asymptomatic patients has also been challenged [63], potentially limiting [99mTc]-DPD use to assessing cardiac involvement in patients already diagnosed with ATTR amyloidosis.

Similar claims have been made about another radio-labeled compound, [99mTc]-PYP. Bokhari et al. proved in a study, which included 25 patients, that [99mTc]-PYP imaging could efficiently discern between ATTR and AL CA [64]. They also developed a standardized [99mTc]-PYP protocol for the diagnosis of ATTR CA [65]. However, larger studies reported significant absorption in patients suffering from AL CA as well. Further studies concerning both tracers would definitely shed some light on their potential use [4].

PET scans with 18F-florbetapir, a tracer originally developed for amyloid imaging in the brain, also represent a promising tool for early diagnosis of cardiac amyloidosis, as well as quantification of cardiac amyloid and extracardiac amyloid load [66]. It must be noted that 18F-florbetapir binds to both AL and ATTR deposits, although a higher affinity for AL amyloid was reported in vitro studies [67]. ^18^F-FDG PET/CT scans are a mainstay of clinical oncology and have also been examined as a potential imaging tool for detecting ATTR and AL amyloidosis. Unfortunately, many organs, including the heart, demonstrated variable avidity to the tracer. This fact limited the scan’s sensitivity of detecting cardiac involvement to 62.5%. ^11^C-Labelled Pittsburgh compound B (^11^C-PiB) is a radio-labeled derivative of thioflavin-T that has been thoroughly used to detect Αβ amyloid deposition in Alzheimer’s disease [68]. The potential application of ^11^C-PiB PET scans in ATTR and AL amyloid imaging was explored, with promising results. The findings of ^11^C-PiB PET tomography correlated well with post-mortem histopathological samples [68]. Further studies are definitely needed to confirm and build upon this work, but the ability to image amyloidosis with ^11^C-PiB would greatly increase PET’s accessibility, as this is a relatively common tracer.

Finally, 123I-MIBG scintigraphy has the capability of detecting cardiac sympathetic denervation in amyloidosis patients with cardiac involvement [69]. Specifically in the case of ATTR CA, 123I-MIBG can return positive results before echocardiographic evidence of disease [70]. Although the increased likelihood of lethal arrhythmias has been proven in the setting of denervation of viable myocardium in patients’ post-myocardial infarction, scarce data exists, with regard to amyloidosis patients. Such a finding would imply that positive 123I-MIBG findings place the patient at heightened risk of arrhythmias.

#### Fat ultrasonography

Misumi et al. introduces fat ultrasonography, a novel tool for the screening and diagnosis of ATTR amyloidosis. The reported sensitivity is 85.1% and the specificity is 97.1%, under ideal imaging conditions [71]. Although the sample size and constitution is not ideal, this study serves as a proof of concept for a technique that constitutes both a potential screening tool a promising research field.

### Biomarkers

#### Cardiac involvement biomarkers

Serum troponin and NT-proBNP, biomarkers classically associated with detecting and evaluating heart failure from causes such as coronary heart disease [72], have proven successful in assessing myocardial involvement in amyloidosis.

Cardiac Troponin T (cTnT) is a reliable marker of cardiomyocyte death and has proven itself a strong negative prognostic factor for overall survival in AL and ATTR amyloidosis [73, 74]. The introduction of a new, high sensitivity assay for measuring cTnT, hs-cTnT, was thought that could improve the staging of AL amyloidosis. It has been shown that although the use of hs-cTnT does not improve the classic Mayo Staging System, it can render NT-ProBNP testing unnecessary, as models indicate that the Staging System retains its strength without it, when hs-cTnT is utilized [73–75].

The Brain Natriuretic Peptide (BNP) and the protein that results from the N-terminal cleavage of BNP’s prohormone, titled NT-proBNP, have been consistently shown to be reliable prognostic markers for cardiac amyloidosis, regardless of the nature of the amyloid (AL or ATTR) [76]. For this reason, NT-proBNP levels have been included in the Mayo Amyloidosis Staging System, which is widely used for the staging of AL amyloidosis [77]. The logarithm of NT-proBNP levels is an independent prognostic factor of mortality [78]. According to the Transthyretin Amyloidosis Outcomes Survey (THAOS), patients with elevated BNP and NT-ProBNP levels at the time of diagnosis demonstrated poorer prognosis, mainly attributed to renal insufficiency and worsened functional status [79].

#### AL amyloidosis-specific biomarkers

Serum and urine immunofixation electrophoresis (IFE), coupled with quantitative free light chain (FLC) measurements, boast an impressive sensitivity in diagnosing monoclonal gammopathies and have become a mainstay in the diagnosis of AL amyloidosis [80]. Recent data suggest that serum IFE alone, combined with FLC only misses 0.5% of monoclonal gammopathy cases with abnormal urinary results [81].

It is of note, that positive FLC and IFE findings in patients with confirmed amyloid deposition, whether through EMB or imaging studies, do not definitively confirm the diagnosis of AL amyloidosis, as cases of older patients with ATTRwt amyloidosis and concomitant MGUS are repeatedly reported throughout the literature. In patients with risk factors for both diseases, diagnostic modalities with the capacity to objectively discern amyloid composition are necessary [82].

dFLC is defined as the difference between the serum levels of κ an λ Free Light Chains. It is a major independent prognostic factor in AL amyloidosis, as well as several other plasma cell disorders such as the multiple and smoldering myeloma [83]. The pathophysiological reasoning is that increased dFLC values reflect more available free light chains, accelerating fibril formation. Increased dFLC levels are associated with higher likelihood and severity of heart disease, as well as worse response to therapy, whilst a reduction post-therapy has been tied to better outcomes [84].

The currently preferred staging system in AL amyloidosis is the Mayo Amyloidosis Staging System that utilizes the aforementioned biomarkers cTNT, NT-proBNP and dFLC to stratify disease severity in patients with AL (Table 1) [77].

**Table 1:** AL Staging according to the revised Mayo AL staging tool [55]

Number of abnormal laboratory tests	Stage (according to revised staging system)	Median Overall Survival (months)	5-year Survival
0	I	94.1	59%
1	II	40.3	42%
2	III	14	20%
3	IV	5.8	14%

(Laboratory tests: cTnT ≥0.025 ng/mL, NT-ProBNP ≥1800 pg/mL, dFLC≥18 mg/dL)

#### ATTR amyloidosis-specific biomarkers

The V122I variant of the TTR protein is being increasingly recognized as an underdiagnosed cause of heart failure in elderly African American patients, 3–4% of which appear to carry the gene [85]. Arvanitis et al. discovered that Retinol Binding Protein 4 (RBP4) levels in ATTRm amyloidosis patients with the V122I mutation have been found significantly diminished [86]. By combining ultrasound measurements (LVEF, IVSD) and ECG parameters such as the mean QRS alongside with serum RBP4 levels, the authors proposed a clinical score with significant diagnostic accuracy [87].

A very similar staging system to that used in AL amyloidosis has been proposed for ATTR-wt amyloidosis, incorporating cTnT and NT-proBNP values to stratify patients in three stages, depending on the number of the aforementioned lab values exceeding a certain cut-off value [88].

Recently, Gillmore et al. devised a novel staging tool that can be used for both ATTRwt and ATTRm amyloidosis (Table 2). They validated its capability to predict median survival in a study consisting of 869 patients. This staging tool assesses eGFR and NT-proBNP, which both correlate well with overall survival [89]. This approach elicited mixed results from the research community. It has been lauded for its simplicity and reproducibility, but criticized for the omission of cTnT from the risk stratification, as well as the inclusion of eGFR. In an insightful editorial, Singh and Falk pointed out that a reduced eGFR in ATTR CA patients is mostly a byproduct of either age-related comorbidities or renal hypoperfusion secondary to heart failure, thus not a good independent prognostic factor of mortality [90]. They also hint towards the general trend of machine learning-based clinical prediction models [91, 92], and their potential application to ATTR patients.

**Table 2:** Proposed ATTR staging utilizing the staging tool proposed by Gillmore et al. [67]

Number of abnormal laboratory tests	Stage (according to revised staging system)	Median Overall Survival (months)	5-year Survival
0	I	69.2	63%
1	II	46.7	37%
2	III	24.1	19%

(Laboratory tests: eGFR< 45 ml/min/1.73 m2, NT-proBNP > 3000 ng/L)

## Conclusion

Cardiac amyloidosis is the main cause of morbidity and mortality in AL and ATTR amyloidosis. Heart failure is almost inevitable during the course of the disease [93–95], greatly limiting therapeutic options. A timely diagnosis is thus critical [96] but frequently proves difficult, as the symptoms are rarely indicative of the disease. The low cost, simplicity and lack of radiation render ultrasonographic protocols an almost ideal tool to “rule in” amyloidosis as a likely cause of congestive heart failure. Less readily available and more expensive tools such as cardiac MRI and nuclear imaging are better used to confirm and quantify cardiac involvement or to screen for it in patients already diagnosed with amyloidosis. Both diagnostic tools have yielded quantitative values (ECV fraction and myocardial retention index respectively) that appear to detect cardiac involvement in amyloidosis patients, so their inclusion in the current staging systems is an interesting avenue of research. These staging systems currently stratify patients solely through the use of biomarkers, whose role in CA is constantly re-evaluated. Current research aims at rendering existing tools more effective and sensitive, in addition to discovering novel disease biomarkers, such as RBP4.

**Fig. 4:**
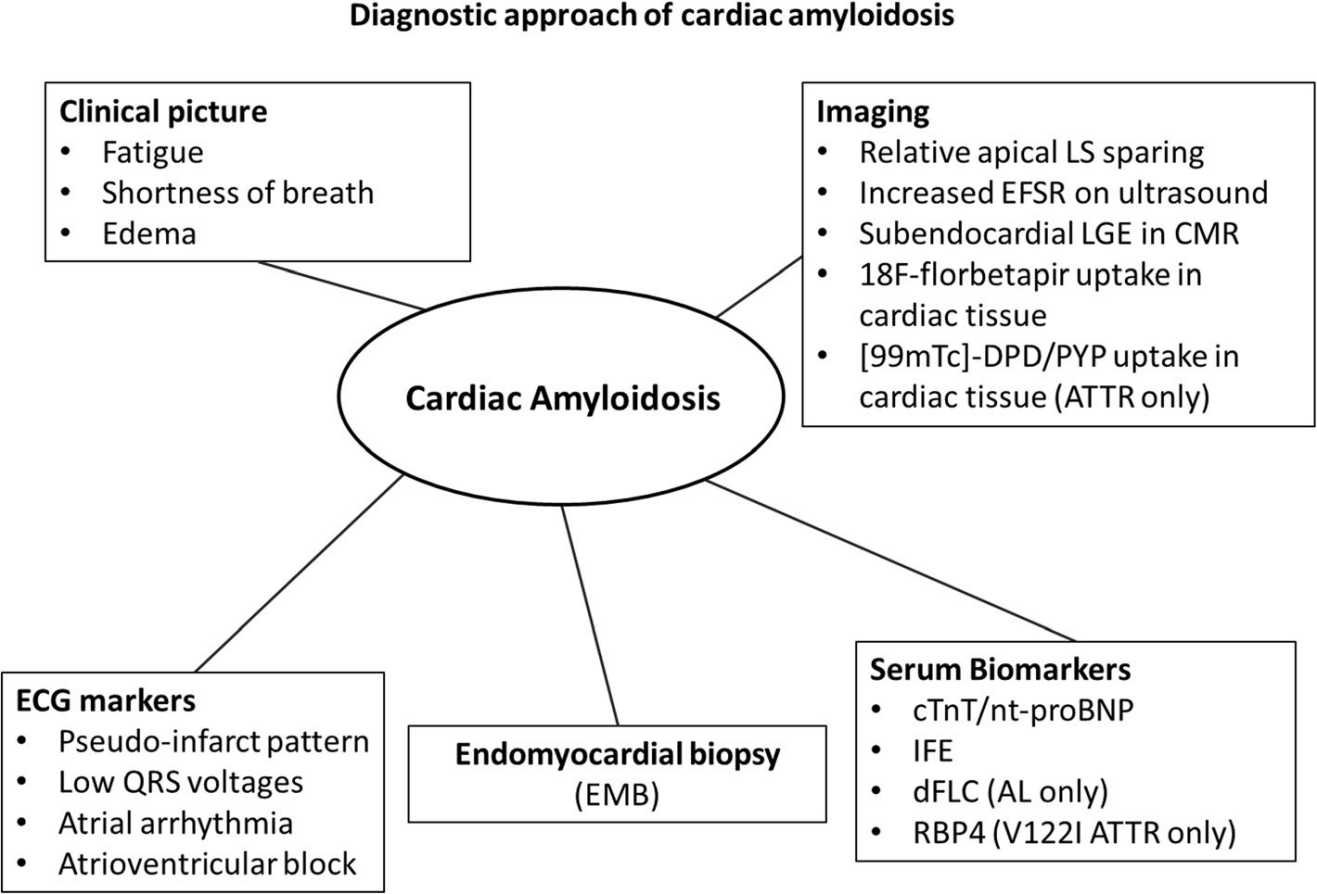
Established diagnostic and staging concepts for patients with cardiac amyloidosis

In summary, several novel diagnostic and staging concepts have been established for cardiac amyloidosis in recent years (Fig. 4). Research is now focusing on validating these novel concepts using larger patient groups and better adjusting them for clinical practice. The validation of diagnostic algorithms that use simplified and cost-effective means of ruling in the diagnosis and sensitive tools to confirm the disease and to quantify amyloid load are key elements to shortening the time to diagnosis and improving patients’ prognosis.

## Abbreviations

[99mTc]-PYP: Tc-99 m-pyrophosphate
123I-MIBG: 123I-metaiodobenzylguanidine
2D: Two-dimensional
2DST: Two-dimensional speckle tracking
3D: Three-dimensional
3DST: Three-dimensional speckle tracking
A: amyloidosis
AL: Light chain amyloidosis
ANP: Atrial Natriuretic Peptide
ATTRm: Mutant Transthyretin amyloidosis
ATTRwt: Wild-type Transthyretin amyloidosis
BNP: Brain Natriuretic Peptide
CA: Cardiac amyloidosis
CMR: Cardiac Magnetic Resonance
cTnT: Cardiac Troponin T
dFLC: Difference in κ and λ light chain concentrations
ECV: Extracellular Volume
EF: Ejection Fraction
EFSR: Ejection Fraction Strain Ratio
eGFR: Estimated Glomerular Filtration Rate
EMB: Endomyocardial biopsy
FLC: Free light chains
GLS: Global Longitudinal Strain
HCM: Hypertrophic Cardiomyopathy
HFpEF: Heart failure with preserved ejection fraction
hs-cTnT: High sensitivity cardiac Troponin T
IFE: Immunofixation electrophoresis
IVSd: Interventricular septal end diastole
LGE: Late Gadolinium Enhancement
LS: Longitudinal strain
LVEF: Left ventricular ejection fraction
LVH: Left ventricular hypertrophy
MRI: Magnetic Resonance Imaging
NT-proBNP: N-terminal pro-Brain Natriuretic Peptide
PSIR: Phase-Sensitive Inversion Recovery
RALS: Relative Apical Longitudinal Strain
RBP4: Retinol binding protein 4
TDI: Tissue Doppler Imaging
THAOS: Transthyretin Amyloidosis Outcomes Survey
TTR: Transthyretin
TTR: Transthyretin[99mTc]-DPD Tc-99 m-3,3-diphosphono-1,2-propanodicarboxylicacid

## References

[1] Maleszewski JJ (2015). Cardiac amyloidosis: pathology, nomenclature, and typing. Cardiovasc Pathol [Internet].

[2] Bayliss M, McCausland KL, Guthrie SD, White MK. The burden of amyloid light chain amyloidosis on health-related quality of life. Orphanet J Rare Dis 2017;12(1):1–10. Available from: doi 10.1186/s13023-016-0564-2 10.1186/s13023-016-0564-2 PMC5244523 28103898

[3] Sperry BW, Vranian MN, Hachamovitch R, Joshi H, Ikram A, Phelan D, Hanna M (2015). Subtype-specific interactions and prognosis in cardiac amyloidosis. J Am Heart Assoc.

[4] Tuzovic M, Yang EH, Baas AS, Depasquale EC, Deng MC, Cruz D, Vorobiof G. Cardiac amyloidosis: diagnosis and treatment strategies. Curr Oncol Rep. 2017;19(7). 10.1007/s11912-017-0607-4 28528458

[5] Dubrey SW, Cha K, Simms RW, Skinner M, Falk RH (1996). Electrocardiography and Doppler echocardiography in secondary (AA) amyloidosis. Am J Cardiol.

[6] Leone O, Boriani G, Chiappini B, Pacini D, Cenacchi G, Martin Suarez S, Rapezzi C, Bacchi Reggiani ML, Marinelli G (2004). Amyloid deposition as a cause of atrial remodelling in persistent valvular atrial fibrillation. Eur Heart J.

[7] Sanders P, Morton JB, Davidson NC, Spence SJ, Vohra JK, Sparks PB, Kalman JM (2003). Electrical remodeling of the atria in congestive heart failure: electrophysiological and electroanatomic mapping in humans. Circulation.

[8] Goette A, Röcken C (2004). Atrial amyloidosis and atrial fibrillation: a gender-dependent “arrhythmogenic substrate”?. Eur Heart J.

[9] Pinney JH, Smith CJ, Taube JB, Lachmann HJ, Venner CP, Gibbs SDJ, Dungu J, Banyperasad SM, Wechalekar AD, Whelan CJ, Hawkins PN, Gillmore JD (2013). Systemic amyloidosis in England: an epidemiological study. Br J Haematol.

[10] Maurer MS, Elliott P, Comenzo R, Semigran M, Rapezzi C (2017). Addressing common questions encountered in the diagnosis and management of cardiac amyloidosis. Circulation.

[11] Fikrle M, Palecek T, Kuchynka P, Nemeciek E, Bauerova L, Straub J, Rysava R (2013). Cardiac amyloidosis: a comprehensive review. Cor Vasa.

[12] Tanskanen M, Peuralinna T, Polvikoski T, Notkola IL, Sulkava R, Hardy J, Singleton A, Kiuru-Enari S, Paetau A, Tienari PJ, Myllykangas L (2008). Senile systemic amyloidosis affects 25% of the very aged and associates with genetic variation in alpha2-macroglobulin and tau: a population-based autopsy study. Ann Med.

[13] González-López E, Gallego-Delgado M, Guzzo-Merello G, De Haro-Del Moral FJ, Cobo-Marcos M, Robles C, Bornstein B, Salas C, Lara-Pezzi E, Alonso-Pulpon L, Garcia-Pavia P (2015). wild-type transthyretin amyloidosis as a cause of heart failure with preserved ejection fraction. Eur Heart J.

[14] Barbero U, Destefanis P (2015). An Indian-look right into restrictive cardiomyopathies. Indian Heart J [Internet].

[15] Rammos Aidonis, Meladinis Vasileios, Vovas Georgios, Patsouras Dimitrios (2017). Restrictive Cardiomyopathies: The Importance of Noninvasive Cardiac Imaging Modalities in Diagnosis and Treatment—A Systematic Review. Radiology Research and Practice.

[16] Westphal JG, Rigopoulos AG, Bakogiannis C, Ludwig SE, Mavrogeni S, Bigalke B, Doenst T, Pauschinger M, Tschöpe C, Schulze PC, Noutsias M (2017). The MOGE(S) classification for cardiomyopathies: current status and future outlook. Heart Fail Rev [Internet].

[17] Ritts AJ, Cornell RF, Swiger K, Singh J, Goodman S, Lenihan DJ (2017). Current concepts of cardiac amyloidosis. Diagnosis, Clinical Management, and the Need for Collaboration Heart Fail Clin.

[18] Priyamvada PS, Srinivas BH, Parameswaran S, Morkhandikar S (2015). Heavy and Light chain amyloidosois presenting as complete heart block: A rare presentation of a rare disease. Indian Journal of Nephrology.

[19] Reisinger J, Dubrey SW, Lavalley M, Skinner M, Falk RH (1997). Electrophysiologic abnormalities in AL (primary) amyloidosis with cardiac involvement. J Am Coll Cardiol [Internet].

[20] Connors LH, Sam F, Skinner M, Salinaro F, Sun F, Ruberg FL, Berk JL, Seldin DC (2016). Heart failure resulting from age-related cardiac amyloid disease associated with wild-type transthyretin: a prospective, observational cohort study. Circulation.

[21] From AM, Maleszewski JJ, Rihal CS (2011). Current status of endomyocardial biopsy. Mayo Clin Proc.

[22] Garcia Yessica, Collins A. Bernard, Stone James R. (2018). Abdominal fat pad excisional biopsy for the diagnosis and typing of systemic amyloidosis. Human Pathology.

[23] Klein AL, Hatle LK, Taliercio CP (1991). Prognostic significance of Doppler measures of diastolic function in cardiac amyloidosis. A Doppler echocardiography study. Circulation.

[24] Holmgren G, Steen L, Suhr O, Ericzon BG, Groth CG, Andersen O, Wallin BG, Seymour A, Richardson S, Hawkins PN, Pepys MB (1993). Clinical improvement and amyloid regression after liver transplantation in hereditary transthyretin amyloidosis. Lancet.

[25] Banypersad SM, Moon JC, Whelan C, Hawkins PN, Wechalekar AD (2012). Updates in cardiac amyloidosis: a review. J Am Heart Assoc.

[26] García-Pavía P, Tomé-Esteban M, Rapezzi C (2011). Amyloidosis. Also a heart disease. Rev Esp Cardiol.

[27] Koyama J, Ray-Sequin PA, Falk RH (2003). Longitudinal myocardial function assessed by tissue velocity, strain, and strain rate tissue doppler echocardiography in patients with AL (primary) cardiac amyloidosis. Circulation.

[28] Koyama J, Davidoff R, Falk RH (2004). Longitudinal myocardial velocity gradient derived from pulsed Doppler tissue imaging in AL amyloidosis: a sensitive Indicator of systolic and diastolic dysfunction. J Am Soc Echocardiogr.

[29] Ha JW, Ommen SR, Tajik AJ, Barnes ME, Ammash NM, Gertz MA, Seward JB, Oh JK (2004). Differentiation of constrictive pericarditis from restrictive cardiomyopathy using mitral annular velocity by tissue Doppler echocardiography. Am J Cardiol.

[30] Cacciapuoti F (2015). The role of echocardiography in the non-invasive diagnosis of cardiac amyloidosis. J Echocardiogr.

[31] Tei C, Dujardin KS, Hodge DO, Kyle RA, Tajik AJ, Seward JB (1996). Doppler index combining systolic and diastolic myocardial performance: clinical value in cardiac amyloidosis. J Am Coll Cardiol.

[32] Sun JP, Stewart WJ, Yang XS, Donnell RO, Leon AR, Felner JM, Thomas JD, Merlino JD (2009). Differentiation of hypertrophic cardiomyopathy and cardiac amyloidosis from other causes of Ventricular Wall thickening by two-dimensional strain imaging echocardiography. Am J Cardiol [Internet].

[33] Quarta CC, Solomon SD, Uraizee I, Kruger J, Longhi S, Ferlito M, Gagliardi C, Milandri A, Rapezzi C, Falk RH (2014). Left ventricular structure and function in transthyretin-related versus light-chain cardiac amyloidosis. Circulation.

[34] Phelan D, Collier P, Thavendiranathan P, Popović ZB, Hanna M, Plana JC, Marwick TH, Thomas JD (2012). Relative apical sparing of longitudinal strain using two-dimensional speckle-tracking echocardiography is both sensitive and specific for the diagnosis of cardiac amyloidosis. Heart [Internet].

[35] Pagourelias ED, Duchenne J, Mirea O, Vovas G, Van Cleemput J, Delforge M, Kuznetsova T, Bogaert J, Voigt JU (2016). The relation of ejection fraction and global longitudinal strain in amyloidosis: implications for differential diagnosis. JACC Cardiovasc Imaging..

[36] Pagourelias ED, Mirea O, Duchenne J, Van Cleemput J, Delforge M, Bogaert J, Kuznetsova T, Voigt J-U. Echo Parameters for Differential Diagnosis in Cardiac Amyloidosis A Head-to-Head Comparison of Deformation and Nondeformation Parameters Circ Cardiovasc Imaging [Internet]. 2017;10(3):e005588. Available from: http://circimaging.ahajournals.org/lookup/doi/10.1161/CIRCIMAGING.116.005588. 10.1161/CIRCIMAGING.116.005588 28298286

[37] Migrino RQ, Harmann L, Woods T, Bright M, Truran S, Hari P (2008). Intraventricular dyssynchrony in light chain amyloidosis: a new mechanism of systolic dysfunction assessed by 3-dimensional echocardiography. Cardiovasc Ultrasound.

[38] Baccouche H, Maunz M, Beck T, Gaa E, Banzhaf M, Knayer U, Fogarassy P, Beyer M (2012). Differentiating cardiac amyloidosis and hypertrophic cardiomyopathy by use of three-dimensional speckle tracking echocardiography. Echocardiography.

[39] Maurer Mathew S., Ruberg Frederick L., Weinsaft Jonathan W. (2018). More Than Meets the Eye: Time for a New Imaging Paradigm to Test for Cardiac Amyloidosis. Journal of Cardiac Failure.

[40] Maceira AM, Joshi J, Prasad SK, Moon JC, Perugini E, Harding I, Sheppard MN, Poole-Wilson PA, Hawkins PN, Pennell DJ (2005). Cardiovascular magnetic resonance in cardiac amyloidosis. Circulation.

[41] Mavrogeni Sophie, Kolovou Genovefa, Bigalke Boris, Rigopoulos Angelos, Noutsias Michel, Adamopoulos Stamatis (2018). Transplantation in patients with iron overload: is there a place for magnetic resonance imaging?. Heart Failure Reviews.

[42] Barbero U, Longo F, Destefanis P, Gaglioti CM, Pozzi R, Piga A (2016). Worsening of myocardial performance index in beta-thalassemia patients despite permanently normal iron load at MRI: a simple and cheap index reflecting cardiovascular involvement?. IJC Metab Endocr [Internet].

[43] Barbero U, Destefanis P, Pozzi R, Longo F, Piga A (2012). Exercise Stress Echocardiography with Tissue Doppler Imaging (TDI) Detects Early Systolic Dysfunction in Beta-Thalassemia Major Patients without Cardiac Iron Overload. Mediterr J Hematol Infect Dis.

[44] Syed IS, Glockner JF, Feng D, Araoz PA, Martinez MW, Edwards WD, Gertz MA, Dispenzieri A, Oh JK, Bellavia D, Tajik AJ, Grogan M (2010). Role of cardiac magnetic resonance imaging in the detection of cardiac amyloidosis. JACC Cardiovasc Imaging..

[45] Falk RH, Quarta CC, Dorbala S (2014). How to image cardiac amyloidosis. Circ Cardiovasc Imaging..

[46] Vogelsberg H, Mahrholdt H, Deluigi CC, Yilmaz A, Kispert EM, Greulich S, Klingel K, Kandolf R, Sechtem U (2008). Cardiovascular magnetic resonance in clinically suspected cardiac amyloidosis. J Am Coll Cardiol [Internet]..

[47] Fontana M, Pica S, Reant P, Abdel-Gadir A, Treibel TA, Banypersad SM, Maestrini V, Bulluck H, Lane TL, Lachmann H, Whelan CJ, Wechalekar A, Manisty C, Herrey AS, Kellman P, Hawkins PN, Moon J (2015). LGE-PSIR is an independent predictor of mortality in cardiac amyloidosis: a 250 patient prospective study. J Cardiovasc Magn Reson.

[48] Fontana M, Pica S, Reant P, Abdel-Gadir A, Treibel TA, Banypersad SM, Maestrini V, Barcella W, Rosmini S, Bulluck H, Sayed RH, Patel K, Mamhood S, Bucciarelli-Ducci C, Whelan CJ, Herrey AS, Lachmann HJ, Wechalekar AD, Manisty CH, Schelbert EB, Kellman P, Gillmore JD, Hawkins PN, Moon JC (2015). Prognostic value of late gadolinium enhancement cardiovascular magnetic resonance in cardiac amyloidosis. Circulation.

[49] Dungu JN, Valencia O, Pinney JH, Gibbs SDJ, Rowczenio D, Gilbertson JA, Lachmann HJ, Wechalekar A, Gillmore JD, Whelan CJ, Hawkins PN, Anderson LJ (2014). CMR-based differentiation of AL and ATTR cardiac amyloidosis. JACC Cardiovasc Imaging.

[50] Di Bella G, Minutoli F, Mazzeo A, Vita G, Oreto G, Carerj S, Anfuso C, Russo M, Gaeta M (2010). MRI of cardiac involvement in transthyretin familial amyloid polyneuropathy. Am J Roentgenol.

[51] Williams LK, Forero JF, Popovic ZB, Phelan D, Delgado D, Rakowski H, Wintersperger BJ, Thavendiranathan P (2017). Patterns of CMR measured longitudinal strain and its association with late gadolinium enhancement in patients with cardiac amyloidosis and its mimics. J Cardiovasc Magn Reson.

[52] Pozo E, Castellano JM, Kanwar A, Deochand R, Castillo-Martin M, Pazos-López P, González-Lengua C, Osman K, Cham M, Cordon-Cardo C, Narula J, Fuster V, Sanz J (2018). Myocardial Amyloid Quantification with Look-Locker Magnetic Resonance Sequence in Cardiac Amyloidosis. Diagnostic Accuracy in Clinical Practice and Histological Validation. J Card Fail.

[53] Fontana M, Banypersad SM, Treibel TA, Maestrini V, Sado DM, White SK, Pica S, Castelletti S, Piechnik SK, Robson MD, Gilbertson JA, Rowczenio D, Hutt DF, Lachmann HJ, Wechalekar AD, Whelan CJ, Gillmore JD, Hawkins PN, Moon JC (2014). Native T1 mapping in transthyretin amyloidosis. JACC Cardiovasc Imaging.

[54] Karamitsos TD, Piechnik SK, Banypersad SM, Fontana M, Ntusi NB, Ferreira VM, Whelan CJ, Myerson SG, Robson MD, Hawkins PN, Neubauer S, Moon JC (2013). Noncontrast T1 mapping for the diagnosis of cardiac amyloidosis. JACC Cardiovasc Imaging [Internet].

[55] Mongeon F-P, Jerosch-Herold M, Coelho-Filho OR, Blankstein R, Falk RH, Kwong RY (2012). Quantification of extracellular matrix expansion by CMR in infiltrative heart disease. JACC Cardiovasc Imaging [Internet]..

[56] Banypersad SM, Sado DM, Flett AS, Gibbs SDJ, Pinney JH, Maestrini V, Cox AT, Fontana M, Whelan CJ, Wechalekar AD, Hawkins PN, Moon JC (2013). Quantification of myocardial extracellular volume fraction in systemic AL amyloidosis: an equilibrium contrast cardiovascular magnetic resonance study. Circ Cardiovasc Imaging.

[57] Treibel TA, Bandula S, Fontana M, White SK, Gilbertson JA, Herrey AS, Gillmore JD, Punwani S, Hawkins PN, Taylor SA, Moon JC (2015). Extracellular volume quantification by dynamic equilibrium cardiac computed tomography in cardiac amyloidosis. J Cardiovasc Comput Tomogr.

[58] Puille M, Altland K, Linke RP, Steen-Müller MK, Klett R, Steiner D, Bauer R (2002). 99mTc-DPD scintigraphy in transthyretin-related familial amyloidotic polyneuropathy. Eur J Nucl Med.

[59] Wizenberg TA, Muz J, Sohn YH, Samlowski W, Weissler AM (1982). Value of positive myocardial technetium-99m-pyrophosphate scintigraphy in the noninvasive diagnosis of cardiac amyloidosis. Am Heart J.

[60] Willerson JT, Parkey RW, Bonte FJ, Lewis SE, Corbett J, Buja LM (1980). Pathophysiologic considerations and Clinicopathological correlates of technetium-99m stannous pyrophosphate myocardial scintigraphy. Semin Nucl Med.

[61] Perugini E (2005). Non-invasive evaluation of the myocardial substrate of cardiac amyloidosis by gadolinium cardiac magnetic resonance. Heart..

[62] Miguel C, De Llorente L, Moral FJDH, González-lópez E, Segovia J, Krsnik I, Miguel C, De Llorente L, Moral FJDH, García-pavía P, González-lópez E, Segovia J, Myocardial IK, Segovia J, Krsnik I, Gonza E (2017). Myocardial uptake of AL amyloidosis Tc-DPD in patients with Myocardial uptake of Tc-DPD in patients with AL amyloidosis. J Am Coll Cardiol.

[63] Lairez O, Pascal P, Victor G, Bastié D, Lavie-Badie Y, Pierre A, Cassol E, Berry I (2017). Bone scintigraphy for cardiac amyloidosis imaging: past, present and future. Médecine Nucléaire [Internet].

[64] Bokhari S, Castaño A, Pozniakoff T, Deslisle S, Latif F, Maurer MS (2013). 99mTc-pyrophosphate scintigraphy for differentiating light-chain cardiac amyloidosis from the transthyretin-related familial and senile cardiac amyloidoses. Circ Cardiovasc Imaging..

[65] Bokhari S, Morgenstern R, Weinberg R, Kinkhabwala M, Panagiotou D, Castano A, DeLuca A, Andrew K, Jin Z, Maurer MS (2018). Standardization of 99mTechnetium pyrophosphate imaging methodology to diagnose TTR cardiac amyloidosis. J Nucl Cardiol [Internet].

[66] Dorbala S, Vangala D, Semer J, Strader C, Bruyere JR, Di Carli MF, Moore SC, Falk RH (2014). Imaging cardiac amyloidosis: a pilot study using 18F-florbetapir positron emission tomography. Eur J Nucl Med Mol Imaging.

[67] Park MA, Padera RF, Belanger A, Dubey S, Hwang DH, Veeranna V, Falk RH, Di Carli MF, Dorbala S (2015). 18F-Florbetapir binds specifically to myocardial light chain and transthyretin amyloid deposits: autoradiography study. Circ Cardiovasc Imaging..

[68] Ezawa N, Katoh N, Oguchi K, Yoshinaga T, Yazaki M, Sekijima Y. Visualization of multiple organ amyloid involvement in systemic amyloidosis using 11C-PiB PET imaging. Eur J Nucl Med Mol Imaging. 2017; Available from: http://link.springer.com/10.1007/s00259-017-3814-1. 10.1007/s00259-017-3814-1 28891012

[69] Hongo M, Urushibata K, Kai R, Takahashi W, Koizumi T, Uchikawa S, Imamura H, Kinoshita O, Owa M, Fujii T (2002). Iodine-123 metaiodobenzylguanidine scintigraphic analysis of myocardial sympathetic innervation in patients with AL (primary) amyloidosis. Am Heart J.

[70] Noordzij W, Glaudemans AWJM, Van Rheenen RWJ, Hazenberg BPC, Tio RA, Dierckx RAJO, Slart RHJA (2012). 123I-labelled metaiodobenzylguanidine for the evaluation of cardiac sympathetic denervation in early stage amyloidosis. Eur J Nucl Med Mol Imaging.

[71] Misumi Y, Ueda M, Yamashita T, Masuda T, Kinoshita Y, Tasaki M (2017). Novel screening for transthyretin amyloidosis by using fat ultrasonography. Ann Neurol.

[72] Maisel A, Krishnaswamy P, Nowak R, McCord J, Hollander J, Duc P, Omland T, Storrow A, Abraham W, Wu H, Wold Knudsen C, Perez A, Kazanegra R, Herrmann H, Mccullough PA (2002). Rapid measurement of b-type natriuretic peptide in the emergency diagnosis of heart failure. N Engl J Med.

[73] Qian G, Wu C, Zhang Y, Chen YD, Dong W, Ren YH (2014). Prognostic value of high-sensitivity cardiac troponin T in patients with endomyocardial-biopsy proven cardiac amyloidosis. J Geriatr Cardiol.

[74] Dispenzieri A, Gertz MA, Kumar SK, Lacy MQ, Kyle RA, Saenger AK, Grogan M, Zeldenrust SR, Hayman SR, Buadi F, Greipp PR, Leung N, Russell SR, Dingli D, Lust JA, Rajkumar SV, Jaffe AS (2014). High sensitivity cardiac troponin T in patients with immunoglobulin light chain amyloidosis. Heart.

[75] Palladini G, Barassi A, Klersy C, Pacciolla R, Milani P, Sarais G, Perlini S, Albertini R, Russo P, Foli A, Bragotti LZ, Obici L, Moratti R, D’Eril GVM, Merlini G (2010). The combination of high-sensitivity cardiac troponin T (hs-cTnT) at presentation and changes in N-terminal natriuretic peptide type B (NT-proBNP) after chemotherapy best predicts survival in AL amyloidosis. Blood.

[76] Lehrke S, Steen H, Kristen AV, Merten C, Lossnitzer D, Dengler TJ, Katus HA, Giannitsis E (2009). Serum levels of NT-proBNP as surrogate for cardiac amyloid burden: new evidence from gadolinium-enhanced cardiac magnetic resonance imaging in patients with amyloidosis. Amyloid.

[77] Kumar S, Dispenzieri A, Lacy MQ, Hayman SR, Buadi FK, Colby C, Laumann K, Zeldenrust SR, Leung N, Dingli D, Greipp PR, Lust JA, Russell SJ, Kyle RA, Rajkumar SV, Gertz MA (2012). Revised prognostic staging system for light chain amyloidosis incorporating cardiac biomarkers and serum free light chain measurements. J Clin Oncol.

[78] Kastritis E, Wechalekar AD, Dimopoulos MA, Merlini G, Hawkins PN, Perfetti V, Gillmore JD, Palladini G (2010). Bortezomib with or without dexamethasone in primary systemic (light chain) amyloidosis. J Clin Oncol.

[79] Kristen AV, Maurer MS, Rapezzi C, Mundayat R, Suhr OB, Damy T, Barroso FA, Rugiero MF, Van Cleemput JJ, Tournev I, Cruz MW, Fine NM, Kristen AV, Schmidt HHJ, Zimmermann T, Gess B, Moelgaard H, Plana JMC, Reines JB, Costello JG, Pavia PG, Blanco JLM, Plante-Bordeneuve V, Adams D, Inamo J, Vita G, Merlini G, Bergesio F, Sekijima Y, Ando Y, Misawa S, Lee GY, Oh J, Briseno MAGD, Hazenberg BPC, Coelho T, Conceicao IM, Maurer MS, Shah SJ, Quan D, Judge DP, Gottlieb SS, Sarswat N, Murali SC, Iyadurai S, Cotts WG, Drachman BM, Dispenzieri A, Steidley DE, Hummel SL, Lenihan DJ, Ventura HO, Jacoby DL, Hoffman JE (2017). Impact of genotype and phenotype on cardiac biomarkers in patients with transthyretin amyloidosis - report from the transthyretin amyloidosis outcome survey (THAOS). PLoS One.

[80] Palladini G, Russo P, Bosoni T, Verga L, Sarais G, Lavatelli F, Nuvolone M, Obici L, Casarini S, Donadei S, Albertini R, Righetti G, Marini M, Graziani MS, D’Eril GVM, Moratti R, Merlini G (2009). Identification of amyloidogenic light chains requires the combination of serum-free light chain assay with immunofixation of serum and urine. Clin Chem.

[81] Katzmann Jerry A., Dispenzieri Angela, Kyle Robert A., Snyder Melissa R., Plevak Matthew F., Larson Dirk R., Abraham Roshini S., Lust John A., Melton L. Joseph, Rajkumar S. Vincent (2006). Elimination of the Need for Urine Studies in the Screening Algorithm for Monoclonal Gammopathies by Using Serum Immunofixation and Free Light Chain Assays. Mayo Clinic Proceedings.

[82] Roof L, Coker WJ, Lazarchick J, Kang Y (2015). Senile transthyretin cardiac amyloidosis in patients with plasma cell dyscrasias: importance of cardiac biopsy for making the correct diagnosis. Aperito J Cell Mol Biol.

[83] Golombick T, Diamond TH, Manoharan A, Ramakrishna R (2012). Monoclonal gammopathy of undetermined significance, smoldering multiple myeloma, and curcumin: a randomized, double-blind placebo-controlled cross-over 4g study and an open-label 8g extension study. Am J Hematol.

[84] Palladini G, Dispenzieri A, Gertz MA, Kumar S, Wechalekar A, Hawkins PN, Schönland S, Hegenbart U, Comenzo R, Kastritis E, Dimopoulos MA, Jaccard A, Klersy C, Merlini G (2012). New criteria for response to treatment in immunoglobulin light chain amyloidosis based on free light chain measurement and cardiac biomarkers: impact on survival outcomes. J Clin Oncol.

[85] Jacobson DR, Alexander AA, Tagoe C, Garvey WT, Williams SM, Tishkoff S, Modiano D, Sirima SB, Kalidi I, Toure A, Buxbaum JN (2016). The prevalence and distribution of the amyloidogenic transthyretin (TTR) V122I allele in Africa. Mol Genet genomic Med.

[86] Arvanitis M, Simon S, Chan G, Fine D, Beardsley P, LaValley M, Jacobson D, Koch C, Berk JL, Connors LH, Ruberg FL (2017). Retinol binding protein 4 (RBP4) concentration identifies V122I transthyretin cardiac amyloidosis. Amyloid.

[87] Arvanitis M, Koch CM, Chan GG, Torres-Arancivia C, LaValley MP, Jacobson DR, Berk JL, Connors LH, Ruberg FL (2017). Identification of transthyretin cardiac amyloidosis using serum retinol-binding protein 4 and a clinical prediction model. JAMA Cardiol.

[88] Grogan M, Scott CG, Kyle RA, Zeldenrust SR, Gertz MA, Lin G, Klarich KW, Miller WL, Maleszewski JJ, Dispenzieri A (2016). Natural history of wild-type transthyretin cardiac amyloidosis and risk stratification using a novel staging system. J Am Coll Cardiol.

[89] Gillmore JD, Damy T, Fontana M, Hutchinson M, Lachmann HJ, Martinez-Naharro A, Quarta CC, Rezk T, Whelan CJ, Gonzalez-Lopez E, Lane T, Gilbertson JA, Rowczenio D, Petrie A, Hawkins PN (2017). A new staging system for cardiac transthyretin amyloidosis. Eur Heart J.

[90] Singh A, Falk RH. ‘A new staging system for cardiac transthyretin amyloidosis’: is it already on the verge of obsolescence? Eur Heart J. 2018:1–3. Available from: http://www.ncbi.nlm.nih.gov/pubmed/29351628%0A. 10.1093/eurheartj/ehx740 29351628

[91] Weng SF, Reps J, Kai J, Garibaldi JM, Qureshi N (2017). Can machine-learning improve cardiovascular risk prediction using routine clinical data?. PLoS One.

[92] Goldstein BA, Navar AM, Carter RE (2017). Moving beyond regression techniques in cardiovascular risk prediction: applying machine learning to address analytic challenges. Eur Heart J.

[93] Falk RH, Comenzo RLSM (1997). The systemic Amyloidoses. New Engl J Med Rev.

[94] González-López E, Gagliardi C, Dominguez F, Quarta CC, De Haro-Del Moral FJ, Milandri A, Salas C, Cinelli M, Cobo-Marcos M, Lorenzini M, Lara-Pezzi E, Foffi S, Alonso-Pulpon L, Rapezzi C, Garcia-Pavia P (2017). clinical characteristics of wild-type transthyretin cardiac amyloidosis: disproving myths. Eur Heart J.

[95] Zhang C, Huang X, Li J (2017). Light chain amyloidosis: where are the light chains from and how they play their pathogenic role? Blood rev.

[96] Palladini G, Merlini G (2016). What is new in diagnosis and management of light chain amyloidosis?. Blood.

